# The History and Capabilities of Herbal Simples

**Published:** 1890-03-08

**Authors:** W. T. Fernie


### The History and Capabilities of Herbal Simples.

By W. T. Fernie, M.D.

*(Continued from page 322.)*

### VI.—THE COMMON BARBERRY—SWEET BASIL.

### The Common Barberry.

The Barberry—Berberis—so called from an Arabian word, grows as a shrub in our English copses and hedges, particularly about Essex. Provincially it is named also Pipperidge bush, from pepin, a pip, and rouge, red, as descriptive of its small, scarlet, juiceless fruit. Its active chemical principles are Berberin and Oxyacanthin. The berries sparingly yield a juice, which is cooling and astringent; this was formerly much esteemed by the Egyptians, when diluted, as a drink in pestilential fevers. Contrarywise, the inner yellow bark of the barberry, which, by reason of its colour, on the doctrine of signatures, has long been supposed to influence the liver, is a biliary purgative. An infusion of this bark, made with boiling light sound ale, and allowed to become cool, is an excellent remedy in jaundice from thickened and retarded bile, as likewise for swollen spleen from malarious exposure. And a medicinal tincture is made with spirit of wine from the root-branches and the root-bark, of which five drops may be given with a tablespoonful of water to an adult, twice or three times in the day. This is highly useful in simple congestion of the liver, with furred tongue, lowness of spirits, and yellow complexion; also for painful urinary gravel associated therewith. British farmers have a great dislike to the barberry shrub, because when it grows in cornfields, the wheat near it is blighted, even to the distance of two or three hundred yards. This is because of a special fungus which often affects the barberry, and being carried by the wind, reproduces itself destructively on the ears of wheat. Among the Italians the barberry bears the name of Holy Thorn, since it is thought to have formed part of the crown of thorns made for our Saviour. From the ripe fruit we make a well known jelly, by boiling the berries with an equal weight of sugar, and then straining. Also a capital syrup is prepared somewhat similarly, with which, when diluted with water, an excellent astringent gargle may be made. Clusius setteth it down as a wonderful secret, which he had of a friend, that if the yellow bark of barberry be steeped in white wine for three hours, and be afterwards drunke, it will purge one very marvellously! The following is a good old receipt for making barberry jam. Pick the fruit from the stalks, and bake it in an earthen pan, then press it through a sieve with a wooden spoon. Having mixed equal weights of the prepared fruit and of powdered sugar, put this in pots and cover it up, then set them in a dry place, having sifted some powdered sugar over the top of each pot.

In Gerard's day, at a village called Ivor, two miles from Colebrooke, most of the hedges consisted of nothing but barberry bushes. When fully blown, the barberry may be taken botanically as a capital text for one of Nature's instructive sermons against the self-fertilisation of flowers. On looking into an open blossom of barberry, the six stamens will be seen lying in ambush at the foot of the six petals, and keeping guard over the honey-bags. Then, if a busy bee comes along intent on rifling the flower of its sweets, the word of command is straightway given "Up, Guards, and at him!" Anon the stamens spring forward directly they are touched by the insect, and having well dusted his jacket with a shower of fructifying pollen, they send the intruder flying to woo the affections of another kindred flower. This pretty fact may be easily verified by touching the base of the stamens in the ripe blossom with the point of a pin.

An amusing little hand-bill now lies before me, which I received lately at one of our most fashionable watering places in the High Street. It belauds the virtues of the barberry as a specific remedy against bile, heartburn, and the black jaundice, which was discovered after infinite pains by one who had studied for thirty years by candle-light for the good of his countrymen.

### Sweet Basil.

The sweet Basil—so called, says Parkinson, because the smell thereof being so excellent it is fit for a king's house—is a herb common in our gardens, but in England it dies down every year, and the seeds have to be sown annually. Botanically it is termed basilicon or royal, probably because used of old in some regal unguent, bath, or medicine. This and the wild Basil, belong to the Labiate order of plants. The latter, Calamintha Clinopodium, or Basil thyme, is a hairy plant, with purple flowers, in whorls, growing in bushy places, about hedges and roadsides, and with a strong odour of cloves. The term Clinopodium signifies bed's foot flower, "because the branches dooe resemble the foot of a bed." The leaves of the sweet Basil when slightly bruised exhale a delightful smell; they gave the famous distinctive flavour to the original Fetter Lane sausages. I do not find that this plant possesses any individual chemical constituent of particular virtue; but, in common with the rest of the Labiates, it furnishes a volatile, aromatic essential oil. On this account it is much employed in France for flavouring soups and sauces; and the dried leaves are used for nervous headaches, in the form of snuff. A tea made by pouring boiling water on the green herb, gently but effectually helps on the retarded monthly flow. We are told that a certain advocate of Genoa was once sent as an ambassador to treat for conditions with the Duke of Milan. The said Duke harshly refused to hear the message, or to grant the conditions. Then the ambassador offered the Duke a handful of Basil. Demanding what this meant, the Duke was told that the properties of the herb were, if gently handled, to give out a pleasant odour; but that, if hardly wrung and bruised, it would breed scorpions. Pleased at this witty answer the Duke confirmed the conditions, and sent the Ambassador honourably home.

Outside its herbal virtues this plant has been made chiefly famous by Keats, in his tender, pathetic poem of Isabella and the Pot of Basil; founded on a story from Boccaccio. "Fair Isabel, poor, simple Isabel," falls deeply in love with Lorenzo, a young palmer; but her proud, avaricious Florentine brothers resent this liberty; and, having treacherously murdered Lorenzo, they bury him in a wood. An avenging Spirit appears to Isabel in a dream, and reveals to her the place where Lorenzo lies decapitated and concealed. She reverently possesses herself of the head, which

"She wrapped up, and for its tomb did choose
A garden-pot, wherein she laid it by,
And covered it with mould, and o'er it set
Sweet Basil, which her tears kept ever wet."

"And so she ever fed it with thin tears,
Whence thick, and green, and beautiful it grew.
So that it smelt more balmy than its peers
Of Basil-tufts in Florence; for it drew
Life from the mouldering head there shut from view."

